# Evaluation of gastric acid suppression with vonoprazan using calcium carbonate breath test

**DOI:** 10.3164/jcbn.18-75

**Published:** 2018-11-28

**Authors:** Shuichi Miyamoto, Momoko Tsuda, Mototsugu Kato, Katsuhiro Mabe, Shuichi Muto, Shoko Ono, Yuichi Shimizu, Naoya Sakamoto

**Affiliations:** 1 Department of Gastroenterology and Hepatology, Hokkaido University Graduate School of Medicine, Kita 14, Nishi 5, Kita-ku, Sapporo, Hokkaido 060-8648, Japan; 2 Department of Gastroenterology, National Hospital Organization Hakodate Hospital, 16-gou, 18-banchi, Kawahara-cho, Hakodate, Hokkaido 041-8512, Japan; 3 Department of Gastroenterology, National Hospital Organization Hokkaido Medical Center, 1-banchi, 1-gou, 5-jo, 7-chome, Yamanote, Nishi-ku, Sapporo, Hokkaido 063-0005, Japan; 4 Division of Endoscopy, Hokkaido University Hospital, Kita 14, Nishi 5, Kita-ku, Sapporo, Hokkaido 060-8648, Japan

**Keywords:** vonoprazan, gastric acid, calcium carbonate breath test, potassium-competitive acid blocker, proton-pump inhibitors

## Abstract

Vonoprazan, a potassium-competitive acid blocker, is a new class of acid-suppressing agent. The acid-inhibitory effect of vonoprazan has been well-documented. However, there is no report on the extent to which the amount of gastric acid secretion is suppressed, not pH measurement, by the use of vonoprazan. The aim of this study was to evaluate this suppression effect. This was a single-arm, interventional pilot study involving 7 healthy Japanese men. The subjects were administered 20 mg vonoprazan for 6 days. The amount of gastric acid secretion was determined using the calcium carbonate breath test. The acid outputs were defined as the maximum Δ^13^C‰ (Δ^13^C max) and area under the curve (AUC) during the 30 min sampling period. The Δ^13^C max and AUC values significantly decreased on the administration of 20 mg vonoprazan. The AUC dropped by approximately 78% on day 1 and by 84% on day 6 and subsequently returned to the control level after cessation of vonoprazan therapy (reduction by 68% on day 7 and by 42% on day 8). In conclusion, the amount of gastric acid secretion rapidly decreased by the administration of vonoprazan; this inhibitory effect was found to be potent and long-lasting. (UMIN ID: UMIN000025469)

## Introduction

The histamine H_2_ (H_2_) receptor antagonists and proton-pump inhibitors (PPIs) are clinically used as gastric acid suppressants worldwide. PPIs strongly inhibit the function of H^+^/K^+^-ATPase in gastric parietal cells and suppress the secretion of gastric acid.^(1)^ These drugs are widely employed for acid-related disorders such as gastric ulcers, duodenal ulcers, and gastroesophageal reflux disease. They are also effective in preventing gastric mucosal injuries resulting from the use of low-dose aspirin and non-steroidal anti-inflammatory drugs as well as in eradicating *Helicobactor pylori* (*H. Pylori*) infection.^(2–5)^ Owing to the well-known and widespread benefits of PPIs, their long-term application has been increasing.^(6)^ However, the use of PPIs could cause the following complications, which need to be looked into: (i) PPIs are acid-activated pro-drugs that convert to gastric acid; therefore, they should be administered before meals to achieve their full efficacy.^(7)^ (ii) The action of PPI is slow and 3–5 days of treatment is usually needed to experience its full efficacy.^(8)^ (iii) PPIs have a short plasma half-life of about 90 min; hence, they are not capable of inhibiting all gastric acid pumps.^(8)^ (iv) Patient’s response to PPIs varies to a great extent because of CYP2C19 metabolism.^(9,10)^ In addition, adverse effects related to long-term use of PPIs, such as fractures,^(11)^ enteric infections,^(12)^ and development of gastric polyps^(13)^ have been reported. Recently, Takagi *et al.*^(14)^ reported a probable association between PPI use and the alternation of microbiota. Vonoprazan is a potassium-competitive acid blocker (P-CAB) that was recently approved for use in Japan (Takeda Pharmaceutical Company Ltd., Tokyo, Japan). P-CAB—a new class of acid-suppressing agents—inhibits gastric H^+^/K^+^-ATPase activity through reversible K^+^-competitive ionic binding to the enzyme. In addition, it does not require acid activation within the parietal cell secretory canaliculus.^(15,16)^ It has been reported that P-CAB is effective in the eradication of *H. pylori* infection^(17)^ and ulcer healing after endoscopic submucosal resection.^(18,19)^ Nishizawa *et al.*^(20)^ reported that the eradication rate of the first-line clarithromycin-based triple therapy with PPIs was significantly lower than that with P-CAB and P-CAB was a better choice of antisecretory agent than PPIs especially in young to middle-aged patients (age ≤50 years). Sakurai *et al.*^(21)^ demonstrated that plasma vonoprazan concentrations peaked at 2 h after dosing. Furthermore, 20 mg vonoprazan has been proven to exhibit a more rapid and sustained acid-inhibitory effect than 20 mg esomeprazole or 10 mg rabeprazole.^(22)^ Nonetheless, there are no reports that suppression effect on the amount of gastric acid secretion. To evaluate this effect, nasogastric tube or endoscope is required for the collection of gastric juice. However, the drawback is that these methods are invasive and complex. Interestingly, some studies have reported that the calcium carbonate breath test (CBT) is useful for estimating changes in gastric acid secretion.^(23,24)^ CBT is a non-invasive test, as the subjects are administered ^13^C-labeled calcium carbonate (Ca^13^CO_3_) orally; subsequently, the amount of ^13^C-labeled carbon dioxide (^13^CO_2_) in the breath (produced upon reaction with the gastric acid) is analyzed.

Ca^13^CO_2_ + 2HCl → CaCl_2_ + H_2_O + ^13^CO_2_

Inada *et al.*^(25)^ reported that a high correlation (r = 0.994) between the ^13^CO_2_ concentration (Cmax) and the total amount of gastric acid in rats with or without PPI. In addition, Shinkai *et al.*^(23)^ established that the maximum Cmax is correlated with the amount of pooled gastric acid in human (r = 0.95). The aim of this study was thus to evaluate the suppressed amount of gastric acid secretion using CBT.

## Subjects and Methods

### Study design

This study was a single-arm, non-randomized, uncontrolled, and interventional pilot study. The research was conducted in accordance with the rules and regulations of the Institutional Review Board at the National Hospital Organization Hakodate Hospital and was registered at the University hospital Medical Information Network (UMIN) Clinical Trials Registry (UMIN ID: UMIN000025469). Moreover, this study complied with the Good Clinical Practice and the Declaration of Helsinki and Japanese regulatory requirements. All subjects provided a written informed consent to participate in the investigation.

### Subjects

Healthy Japanese men aged 20–45 years, weighing ≥50.0 kg, with a body mass index (BMI) of ≥18.5 and <30.0 kg/m^2^, who tested negative for *H. pylori* and did not exhibit any gastric atrophic changes (as confirmed by endoscopy), were found eligible for inclusion in the study.

### Blood samples

The fasting blood samples were collected from the subjects on day 0 and on day 6. These samples were immediately centrifuged at 4°C, and the serum was stored at −20°C. The serum gastrin levels were determined using a radioimmunoassay kit (Gastrin RIA Kit II, Fujirebio Inc., Tokyo, Japan), the pepsinogen (PG) I and II levels were quantified by chemiluminescent immunoassay (Lumipulse Presto Pepsinogen I and II kit, Fujirebio Inc., Tokyo, Japan).

### Determination of *Helicobacter pylori* infection status

The presence of *H. pylori* infection was diagnosed by the urea breath test (Otsuka Pharmaceutical Co., Ltd., Tokyo, Japan). The endoscopic findings of regular arrangement of collecting venules in the gastric angle suggested the absence of *H. pylori* infection in the gastric mucosa.^(26)^

### Assessment of gastric atrophy

Endoscopic atrophy was defined according to the Kimura–Takemoto classification system, in which the atrophic patterns are divided into either closed type (C-0, C-1, C-2, and C-3) or open type (O-1, O-2, and O-3) based on the endoscopically recognized differences in the color and height of the gastric mucosa. The lack of endoscopic atrophy was referred to as type C-0 in the Kimura and Takemoto classification.^(27)^

### Calcium carbonate breath test

The subjects were administered with a single oral dose of 125 mg Ca^13^CO_3_ (Cambridge I sotope Laboratories, Inc, MA) suspended in 200 ml water and were maintained in an upright position during the breath test. The breath samples were collected in breath collection bags before receiving the dose as well as at 5, 10, 15, 20, and 30 min after the dosing. At each instance, the ^13^CO_2_ levels in the expired breath were measured using an infrared spectral analyzer (POCone; Otsuka Electronics Co., Ltd., Hirakata, Osaka, Japan), and the change (Δ^13^C‰) from the baseline level was calculated.

### Assessment of acid output

The orally administered Ca^13^CO_3_ reacted with gastric acid in the stomach and produced ^13^CO_2_. The ^13^CO_2_ levels in the expired breath were determined at each breath-sampling time, before and after the administration of Ca^13^CO_3._ The ^13^CO_2_/^12^CO_2_ ratio was calculated each time, and the change (Δ^13^C‰) from the baseline level was also determined. The Δ^13^C‰ in each breath test was plotted as a time curve. The acid outputs were defined as the maximum Δ^13^C‰ (Δ^13^C max) and area under the curve (AUC) during the 30-min sampling period.

### Study protocol

**Fig. 1:**
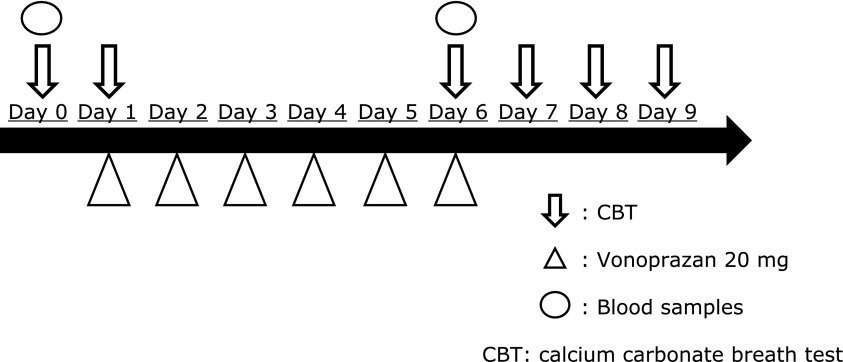
Study protocol. Subjects administered with 20 mg vonoprazan after breakfast (07:00 AM) from day 1 to day 6. The gastric acid secretion amount was examined by the calcium carbonate breath test (CBT) on days 0, 1, 6, 7, 8, and 9 (that is, 5 h after vonoprazan administration or at 12:00 AM). The blood samples were obtained on days 0 and 6.

The subjects were administered 20 mg vonoprazan after breakfast (07:00 AM) from day 1 to day 6 (Fig. 1). The amount of gastric acid secretion was examined by CBT on days 0, 1, 6, 7, 8, and 9 (5 h after vonoprazan administration or at 12:00 AM). The blood samples were obtained on days 0 and 6. The subjects were not permitted to take prescription medications, vitamin supplements, nutrient supplements, or over-the-counter drugs for 28 days before day 0 as well as throughout the duration of the study until day 9. All the subjects consumed an identical breakfast comprising of a rice ball at 07:00 AM and refrained from eating after 09:00 PM during the entire study period.

### Statistical analyses

The results were entered into a database for statistical analysis using Prism software (ver. 6; GraphPad Software, Inc., La Jolla, CA). The data were expressed as mean ± SD. The parameters were compared by Student’s *t* test, and the differences were considered to be statistically significant at *p*<0.05.

## Results

### Subjects

A total of 7 male volunteers were enrolled in this study. None of them displayed gastric atrophic change or active *H. pylori* infection. The median age of the subjects was 33 and the median BMI was 22.7 kg/m^2^ (Table 1).

### Suppression of gastric acid secretion and its examination by CBT

**Fig. 2:**
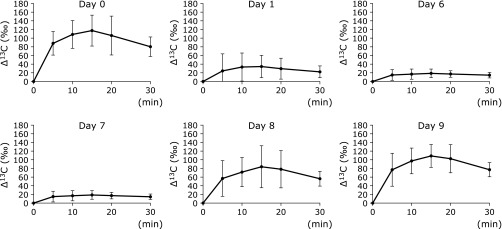
The mean Δ^13^C in expired breath air versus time curve. This graph shows the mean Δ^13^C (‰) time curves after oral administration of 125 mg Ca^13^CO_3_ (*n* = 7). Data are expressed as mean ± SD. *p<0.05 vs day 0. **p<0.01 vs day 0.

**Fig. 3:**
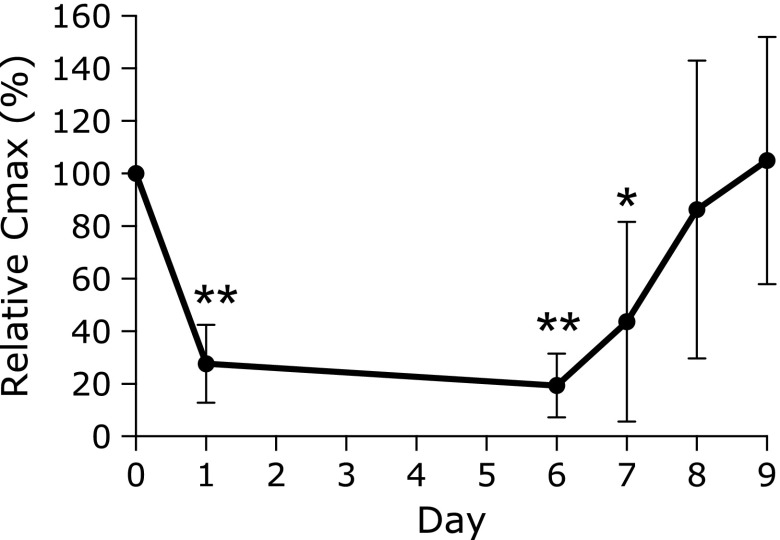
Changes in the relative mean Δ^13^C maximum of Δ^13^CO_2_ versus time curves. This graph shows the changes in the relative Δ^13^C (‰) maximum (Δ^13^C max) (*n* = 7). Data are expressed as mean ± SD. *p<0.05 vs day 0. **p<0.01 vs day 0.

The mean Δ^13^CO_2_ versus time curves are depicted in Fig. 2. The Δ^13^CO_2_ was significantly decreased on day 1, and this low level was maintained until day 6 (last day of vonoprazan administration) and day 7 (cessation of vonoprazan administration). Subsequently, the Δ^13^CO_2_ level gradually returned to the control value on days 8 and 9, that is, after the cessation of vonoprazan administration. Relative Δ^13^C max is illustrated in Fig. 3. The relative Δ^13^C max value significantly decreased on day 1, day 6 (*p*<0.01), and day 7 (*p*<0.05). Subsequently, the relative Δ^13^C max gradually returned to the control levels.

**Fig. 4:**
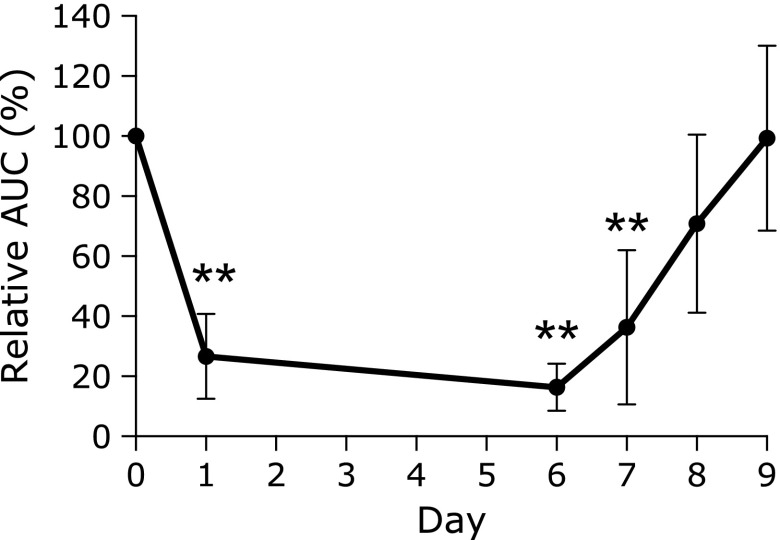
Changes in the relative mean area under the curve of Δ^13^CO_2_ versus time curves. This graph shows changes in the relative area under the curve (AUC) (*n* = 7). Data are expressed as mean ± SD. **p<0.01 vs day 0.

The relative AUC is depicted in Fig. 4. The AUC dropped by approximately 78% on day 1 and by approximately 84% on day 6 compared with day 0. Later, AUC gradually returned to the control level after the cessation of vonoprazan administration (reduction of 68% on day 7 and 42% on day 8). The respective AUCs are indicated in Table 2. Half of the control AUC on day 8 (2 days after cessation of vonoprazan) was not achieved by 3 of the 7 cases. In addition, one of the subjects did not reach the control AUC level even on day 9 (3 days after cessation of vonoprazan).

### Serum gastrin and pepsinogen levels

**Fig. 5:**
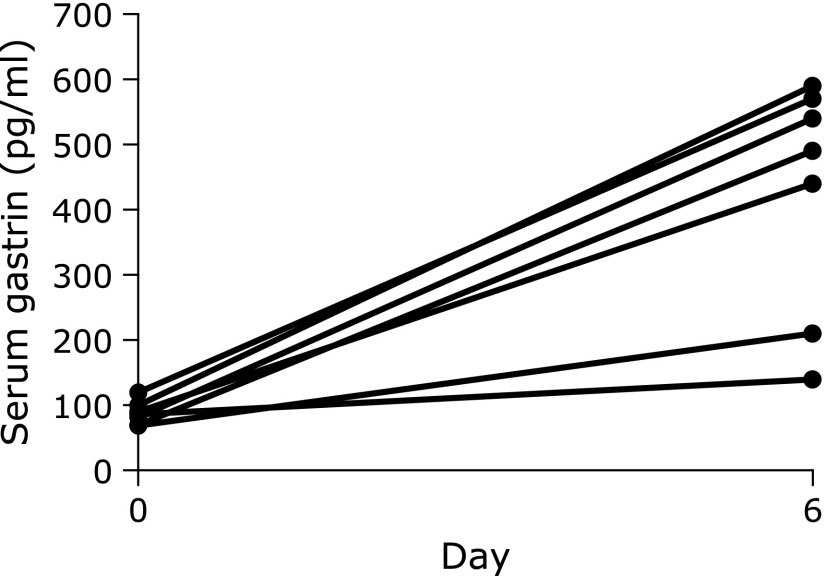
Serum gastrin level. This graph shows the level of serum gastrin (pg/ml) on day 0 (control) and day 6 [5 days after continuous dosing of vonoprazan (20 mg/day)] (*n* = 7).

**Fig. 6:**
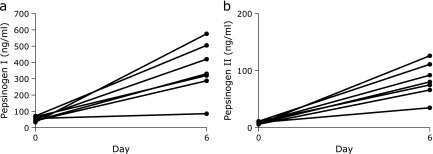
Serum pepsinogen I and pepsinogen II levels. (a) This graph shows the level of serum pepsinogen I (ng/ml) (PG I) on day 0 and day 6 [5 days after continuous dosing of vonoprazan (20 mg/day)] (*n* = 7). (b) This graph shows the level of serum pepsinogen II (ng/ml) (PG II) on day 0 and day 6 [5 days after continuous dosing of vonoprazan (20 mg/day)] (*n = *7).

The results of serum gastrin level examination are given in Fig. 5. The median level of control serum gastrin was 86 pg/ml on day 0. The median level was significantly increased on day 6 (*p*<0.01), and the median level was 540 pg/ml. However, in 2 of the 6 cases, the levels were only slightly increased. The results of serum PG I and PG II levels are given in Fig. 6. The median levels of serum PG I on day 6 was significantly increased (*p*<0.01). However, a subject who showed a slight increase in the level. Similarly, the median level of serum PG II on day 6 was significantly increased (*p*<0.01).

## Discussion

This is the first study to evaluate the suppression and recovery of gastric acid secretion during administration and withdrawal of vonoprazan using CBT. We confirmed that vonoprazan suppressed approximately 80% gastric acid secretion starting from 5 h after the dosing up to 6 days of continuous dosing. In addition, the effect of vonoprazan was continuous, with the reduction of 68% on day 7 (that is 1 day and 5 h after cessation of dosing) and 42% on day 8 (that is 2 days and 5 h after cessation of dosing).

In this study, we evaluated the gastric acid secretion 5 h after the dosing of 20 mg vonoprazan. It has been previously reported that the plasma vonoprazan concentration peaks at 2 h after dosing^(21,28)^ and that the intragastric pH peaks at 4–5 h after dosing.^(22)^ We found that the amount of gastric acid secretion reduced by approximately 80% at 5 h after dosing with 20 mg vonoprazan. Sakurai *et al.*^(22)^ revealed that the intragastric pH level within 24 h tended to be higher after vonoprazan administration than that after esomeprazole or rabeprazole administration. The amount of gastric acid secretion decreased rapidly on the administration of vonoprazan. This study thus ascertained that the gastric acid suppression by the use of vonoprazan continued for at least 2 days after cessation of the dosing, with 68% reduction at 29 h after cessation of dosing and 42% reduction at 53 h after cessation of dosing. The elimination half-life of vonoprazan was up to 9 h.^(21)^ In previous reports, the acid secretion inhibitory effect (pH>6) was sustained for almost 24 h by vonoprazan.^(29)^ Therefore, these results showed that the gastric acid secretion inhibitory effect of vonoprazan is potent and long-lasting. The reason behind this phenomenon, however, remains unknown. The binding of vonoprazan to the proton pump is reversible and competitive, unlike the irreversible binding of PPI. There is a possibility that the acid resistance of activated vonoprazan and its accumulation in parietal cells and secretory canaliculus is related to remnant acid suppression.^(30,31)^ Furthermore, there is a probability of individual differences occurring in the acid secretion inhibitory effect after cessation of dosing. In 2 subjects, the effect was stronger than that in others. Vonoprazan is metabolized mainly by cytochrome CYP3A4.^(32,33)^ Sata *et al.*^(34)^ documented the existence of a mutant variant of CYP3A4. There are a few reports on the clinical effects of vonoprazan by the variant of CYP3A4.^(32,35,36)^ In this study, the variant of CYP3A4 was not examined. This is possibly due to the effect exerted by the variant of CYP3A4.

Recently, there were reported that some factors associated with P-CAB non-responder (non-improvement of symptoms) in the patients with PPI-refractory GERD such as sleep disturbances, co-existing functional dyspepsia and alcohol abstinence.^(37)^ However, there was not reported that factors of P-CAB non-responder (non-suppression of gastric acid) other than CYP3A4.

There were reported that H_2_ receptor antagonists and PPIs had a post-treatment rebound acid hypersecretion.^(38,39)^ In this study, the AUC of Δ^13^CO_2_ on day 9 (3 days after cessation of vonoprazan) was higher than day 0 (before administration of vonoprazan) in the 5/7 cases, however there was no significant change. Although the rebound phenomena by P-CAB has not been reported, there is a possibility of the rebound phenomena by P-CAB.

It has been reported that hypergastrinemia induced by vonoprazan was greater than that induced by PPI. In the present study, the mean serum gastrin level was 540 pg/ml on day 6 of 20 mg vonoprazan administration. Suzuki *et al.*^(40)^ reported that the mean serum gastrin level was 504 pg/ml on day 7 of 20 mg vonoprazan administration. Therefore, the serum gastrin levels are rapidly increased with the use of 20 mg vonoprazan. In this study, the serum gastrin levels of 5/7 cases were over 400 pg/ml on Day 6. On the other hand, the serum gastrin levels of case 1 was 140 pg/ml on day 6 and that of case 4 was 210 pg/ml. However, the AUC of Δ^13^CO_2_ on day 6 of these 2 cases dropped as with another 5 cases. It has been reported that an elevated gastrin (>100 pg/ml) was associated with gastric acid suppression (gastric pH<4 for 50% of time) in patients with use of PPI.^(41)^ In these 2 cases, serum gastrin levels were slightly elevated (>100 pg/ml). The cause of differences of elevated gastrin levels is unclear, however there is possibility of individual differences. An increased risk of carcinoid tumor development has been reported with the long-term use of PPIs for hypergastrinemia.^(42,43)^ However, no development of carcinoid tumor with the long-term use of vonoprazan has been reported. There is therefore a need to follow-up esophagogastroduodenoscopy in patients using vonoprazan over a long-term.

In this study, we evaluated the changes in gastric acid secretion by CBT, which is a non-invasive test used to calculate gastric acid secretion. The subjects took only oral Ca^13^CO_3_ and the procedure did not involve the use of nasogastric tubes or endoscopes for collecting the gastric juices. It was reported that CBT well correlated with the total amount of gastric acid calculated by multiplying the gastric acidity by the volume of gastric juice.^(25)^ Therefore, CBT is useful for calculating gastric acid secretion.

The limitations of the present study include the fact that it was a single-center study with a small sample size. In addition, the CYP3A4 mutation was not examined.

In conclusion, the gastric acid secretion was rapidly decreased by dosing with vonoprazan, and this gastric acid secretion inhibitory effect of vonoprazan was potent and long-lasting.

## Author Contributions

SM, MT and MK conceived the study, designed and executed experiments, analyzed data, prepared figures and tables, and wrote the manuscript. KM, SM, SO, YS and NS supervised all aspects of the study.

## Figures and Tables

**Table 1:** Subjects

Case	Age	BMI	UBT	Atrophy	RAC	*H. pylori* infection
1	33	19.6	0.1	(–)	(+)	(–)
2	40	23.5	0.1	(–)	(+)	(–)
3	32	23.5	0.3	(–)	(+)	(–)
4	34	22.7	0.8	(–)	(+)	(–)
5	27	20.2	0.7	(–)	(+)	(–)
6	31	20.5	0.7	(–)	(+)	(–)
7	44	28.7	0.2	(–)	(+)	(–)

BMI, body mass index (kg/m^2^); UBT, urea breath test; RAC, regular arrangement of collecting venule; *H. pylori*, *Helicobacter pylori*.

**Table 2:** AUC of Δ^13^CO_2_ vs time curves

Case	Day 0	Day 1	Day 6	Day 7	Day 8	Day 9
1	1,493.5	358.75	89.25	172	796	1,842.75
2	2,841.75	623.5	456.25	885.25	2,055.25	2,753.75
3	2,565.25	254.25	679.25	1,937	3,040.25	3,260.75
4	3,932.75	2,067.75	382.75	676.5	1,863.75	2,161.25
5	2,220.5	431.75	300.75	779	1,391.75	2,472.5
6	2,360	479.75	391.5	1,605.5	2,436.5	2,484
7	2,431	926.25	641.5	391.5	931.5	2,850

## Abbreviations

AUC: area under the curve
BMI: body mass index
Ca^13^CO_3_: ^13^C-labeled calcium carbonate
CBT: calcium carbonate breath test
P-CAB: potassium-competitive acid blocker
PPIs: proton-pump inhibitors
